# Explainability in medicine in an era of AI-based clinical decision support systems

**DOI:** 10.3389/fgene.2022.903600

**Published:** 2022-09-19

**Authors:** Robin L. Pierce, Wim Van Biesen, Daan Van Cauwenberge, Johan Decruyenaere, Sigrid Sterckx

**Affiliations:** ^1^ The Law School, University of Exeter, Exeter, United Kingdom; ^2^ Head of Department of Nephrology and Centre for Justifiable Digital Healthcare, Ghent University Hospital, Ghent, Belgium; ^3^ Centre for Justifiable Digital Healthcare, Ghent University Hospital, Ghent, Belgium; ^4^ Department of Intensive Care Medicine and Centre for Justifiable Digital Healthcare, Ghent University Hospital, Ghent, Belgium; ^5^ Department of Philosophy and Moral Sciences, Bioethics Institute Ghent, Ghent University, Ghent, Belgium

**Keywords:** transparency, semantic transparency, artificial intelligence in medicine, clinical decision support, causality, explainability

## Abstract

The combination of “Big Data” and Artificial Intelligence (AI) is frequently promoted as having the potential to deliver valuable health benefits when applied to medical decision-making. However, the responsible adoption of AI-based clinical decision support systems faces several challenges at both the individual and societal level. One of the features that has given rise to particular concern is the issue of explainability, since, if the way an algorithm arrived at a particular output is not known (or knowable) to a physician, this may lead to multiple challenges, including an inability to evaluate the merits of the output. This “opacity” problem has led to questions about whether physicians are justified in relying on the algorithmic output, with some scholars insisting on the centrality of explainability, while others see no reason to require of AI that which is not required of physicians. We consider that there is merit in both views but find that greater nuance is necessary in order to elucidate the underlying function of explainability in clinical practice and, therefore, its relevance in the context of AI for clinical use. In this paper, we explore explainability by examining what it requires in clinical medicine and draw a distinction between the function of explainability for the current patient versus the future patient. This distinction has implications for what explainability requires in the short and long term. We highlight the role of transparency in explainability, and identify semantic transparency as fundamental to the issue of explainability itself. We argue that, in day-to-day clinical practice, accuracy is sufficient as an “epistemic warrant” for clinical decision-making, and that the most compelling reason for requiring explainability in the sense of scientific or causal explanation is the potential for improving future care by building a more robust model of the world. We identify the goal of clinical decision-making as being to deliver the best possible outcome as often as possible, and find—that accuracy is sufficient justification for intervention for today’s patient, as long as efforts to uncover scientific explanations continue to improve healthcare for future patients.

## 1 Introduction

The combination of “Big Data” and Artificial Intelligence (AI) is frequently promoted as being likely to offer health benefits when applied to medical decision-making (e.g., Fogel and Kvedar 2018; Topol 2019). However, many have rightly observed that AI does not automatically transform data into improved health outcomes (e.g., Beam and Kohane, 2018; Emanuel and Wachter 2019). This technology comes with associated risks, not only at the societal level, but also at the levels of individual patient health and physician responsibility and liability. Moreover, the possibilities for bias, for example, because of a limited appreciation of the clinical context and unintended consequences, for example de-skilling, abound (Cabitza et al., 2017).

One of the features of AI that has garnered considerable attention is the issue of *explainable* AI (London 2019; Lauritsen et al., 2020; Duran and Jongsma 2021; Markus et al., 2021). For many, a basic concern is that if the way an algorithm arrives at a particular output is not known (or knowable) by the physician, this lack of explainability may have an impact on the ability to assess the appropriateness and merits of an output designed to inform treatment or diagnosis. As a consequence, this may also jeopardize the quality of the actual medical decision, as well as the shared decision-making process with the patient. There is however still no consensus on the meaning of “explainability” in the context of AI for clinical decision support systems (CDSS), and even less agreement on what kind of “explainability” is required to adequately address such considerations and for responsible adoption of CDSS (e.g., Adadi and Berrada 2018; Payrovnaziri et al., 2020). In general terms, advocates of the central role of explainability in AI base their view on some version of the argument that “certain actions are morally unjustified given the lack (of) the *epistemic warrants* required for the action to take place,” and in the particular context of clinical medicine this implies that “physicians require their beliefs to be epistemically justified before acting,” hence “(a) physician is not morally justified in giving a certain treatment to a patient unless the physician has *reliable knowledge* that the treatment is likely to benefit the patient” (Duran and Jongsma 2021: 331-332, emphasis added)^1^. However, the question of what constitutes “reliable knowledge,” both conceptually and procedurally, such that it provides epistemic justification, remains elusive. If we understand “reliable knowledge,” as used in this context, to refer to a sufficient basis for making an ethically defensible decision in the clinical context, then the term should also point out what should be required of explainability in the use of AI. The relevance of the debate on opacity versus transparency for regulators is also clear from the recent *Proposal for a Regulation of the European Parliament and of the Council Laying down Harmonised Rules on Artificial Intelligence* (European Commission 2021).

Many commentators (e.g., Rudin, 2021; Van Calster et al., 2019; Shortliffe and Sepulveda 2018) worry that the opaque nature of the decision-making of many AI systems implies that, in the specific context of clinical medicine, physicians and patients cannot and should not rely on the results of such systems. In contrast, some strongly oppose the central role of explainability in AI. These commentators argue that there is no reason to require of AI that which is not required of physicians, and emphasize that a lack of explainability does not necessarily hinder a responsible and effective practice of medicine. For example, philosopher Alex London (2019: 17) claims that, much of the time, physicians cannot explain why they are doing things the way they do, and that their interventions are thus also opaque: “(Medicine’s) knowledge of underlying causal systems is in its infancy … Medicine is a domain in which the ability to intervene effectively in the world by exploiting particular causal relationships often derives from experience and precedes our ability to understand why interventions work.” Veliz et al. (2021) also note that many ill-understood processes have been adopted in medicine. One example that is frequently mentioned is the use of aspirin. Physicians did not exactly know how it works, but they knew that, for certain maladies, it did work and reliably so. However, Veliz et al. (2021) rightly point out that we need to investigate the differences and similarities between opaque algorithms and medical treatments whose workings are opaque: “For starters, the mechanism of aspirin is constant over time, but many black-box algorithms change as they get new information. Furthermore, how aspirin works is a natural fact; how algorithms work depends on us.” (Veliz et al., 2021: 340).

Whether it is appropriate to expect “more” explainability from medical AI systems than from physicians is a complicated matter. In London’s (2019) view, put simply, it may be unnecessary to expect explainability from medical AI, since accuracy may well be enough in many cases, even if the “why” or “how” cannot be explained or understood. This point is powerfully illustrated by the fact that the consumption of citrus fruits by sailors to prevent scurvy probably saved thousands of lives, as demonstrated in the first ever RCT, despite the fact that it was then unknown how and why it worked^2^.

Thus, for some, adequately addressing the feature of “opaqueness” appears to be central to identifying what would constitute responsible use of AI in medicine, whereas, for others, it serves little practical purpose.

We see merit in both positions, but in our paper we seek to show that greater nuance is needed in order to get at the underlying function of explainability from the point of view of clinical practice. We aim to contribute to answering the questions which phenomena can be understood as explainability in the context of AI for clinical decision support systems and what kind of “explainability” is required for responsible adoption of such CDSS. While such an analysis provides some insight into *what may be required*, the question begs clarification about the actual value and, hence, *importance* of explainability. To this end, we will explore some key criteria identified in the literature and evaluate whether they are indeed necessary conditions of explainability in a clinical context. First, we will explore whether the concept of transparency furthers explainability. Next, we take up the question of whether accuracy and performance of the AI provide an acceptable form of explanation, and whether this would be sufficient to claim that the AI device is explainable in the sense that it provides a necessary epistemic justification for responsible use in a clinical context. Finally, based on our inquiry we will evaluate whether CDSS currently are able to meet these criteria. Thus, our inquiry into explainability in this paper is focused on the extent to which explainability should be required for AI systems intended for supporting clinical decision-making by physicians and, if so, how this concept of explainability should be understood^3^.

Clinical decision support (CDS) can be defined as a process that “provides clinicians, staff, patients, or other individuals with knowledge and person-specific information, intelligently filtered or presented at appropriate times, to enhance health and health care” (Osheroff et al., 2007: 141)^4^. As noted by Musen et al. (2014):

“Systems that provide CDS do not simply assist with the retrieval of relevant information; they communicate information that takes into consideration the particular clinical context, offering situation-specific information and recommendations. At the same time, such systems do not themselves perform clinical decision making; they provide relevant knowledge and analyses that enable the ultimate decision makers—clinicians, patients, and health care organizations—to develop more informed judgments … Systems that provide CDS come in three basic varieties: 1) They may use information about the current clinical context to retrieve highly relevant online documents, as with so-called “infobuttons” …; 2) they may provide patient-specific, situation-specific alerts, reminders, physician order sets, or other recommendations for direct action; or 3) they may organize and present information in a way that facilitates problem solving and decision making, as in dashboards, graphical displays, documentation templates, structured reports, and order sets” (Musen et al., 2014: 643-644).

The paper proceeds with Section 2, in which we discuss key conceptual issues necessary to clarify the debate on explainability. We particularly focus on the differences between “explainability” and “transparency,” and highlight the crucial importance of *semantic* transparency, as a particular form of transparency that is essential to responsible use of CDSS. Semantic transparency yields a type of explainability that is frequently necessary for accuracy. The clinical case of Acute Kidney Injury (AKI) is provided as an illustration of the importance of this semantic transparency.

In Section 3, we discuss the reasons for why explainability matters in clinical medicine and thus why we need explainability in CDSS. Here, we will build on philosopher of science and technology Duran’s (2021) argumentation for the importance of *scientific* explanation for clinical uses of AI and illustrate this with the example of a prediction model for AKI. Based on this analysis, we argue that the need for scientific or causal explainability in *clinical practice* is limited and that a nuanced approach that engages with the function (and relative importance) of explainability is necessary in order to identify what should be required of medical AI. We argue that, in daily clinical practice, it is sufficient most of the time to have an explanation that provides enough justification to (not) do something, but that, in order to improve accuracy in the longer term, increasing understanding of underlying causality is required^5^.

Section 4 then focuses on this topic of causal understanding, identifying the key question of whether the Big Data approaches that typically underpin modern CDSS can answer questions pertaining to causality (counterfactual or “why” questions). We provide a brief overview of the intense debate on this question, highlighting philosopher of science and technology Pietsch’s (2021) epistemological analysis of Big Data. Pietsch’s (2021) argues that causal understanding is crucial for reliable predictions as well as for effective interventions. We add nuance to his argument on two points: first, that when the accuracy of predictive algorithms operates in the place of explainability, there is no real need for an underlying causal relationship between the data and the outcome; and second, that relying on “variational evidence” allows one to infer a *causal* relationship between a phenomenon and its circumstances. We do not subscribe to the latter—i.e., the claim that causality can be obtained with Big Data approaches relying on machine learning—because, as in many other real-world problems, in a clinical context it is almost never certain that Big Data are complete and representative of all conditions, hence the conditions that would allow for the use of variational induction are simply never present.

In Section 5, we conclude with a summary of our core findings regarding what explainability requires for responsible clinical decision-making.

## 2 “Explainability” and “transparency”: The importance of semantic transparency

The relationship between “explainability” and “transparency” is neither obvious nor clear-cut. Providing one does not necessarily ensure the other. If explainability could be substituted by transparency, then the requirements for explainable AI would be simplified considerably. However, like many commentators, we hold the view that transparency is of limited value as a surrogate for explainability. (Markus et al., 2021: 3). Nevertheless, we will identify a particular type of transparency, semantic transparency, as fundamental to explainability. This, in turn, informs our argument about the nature of the explainability that may ultimately be required.

According to Duran and Jongsma (2021), the concept of “transparency” refers to “algorithmic procedures that make the inner workings of a black box algorithm interpretable to humans” (Duran and Jongsma 2021: 330). In contrast with “transparency,” “opacity” refers to the “inherent impossibility of humans to survey an algorithm, both understood as a script as well as a computer process” (ibid.)^6^.

Duran and Jongsma (2021) give a clear and helpful explanation of why *transparency*, i.e., providing exogenous algorithms capable of making visible the variables and relations within the black box that are responsible for the outcome, although it can help foster trust in algorithms and their outcomes, but does not answer (all) the problems posed by opacity, as it instead *shifts the question of opacity of the black box algorithm to the question of opacity of those* exogenous algorithms.

According to Duran and Jongsma (2021), those defending the view that “epistemic opacity” is inevitable argue that this is due to the fact that humans are limited cognitive agents and that therefore we should abandon the goal of achieving transparency as a means of cultivating trust in algorithms^7^. Duran and Jongsma (2021), by contrast, argue that “giving up explanation altogether (or reducing explanation to a handful of alleged transparent algorithms) defeats much of the purpose of implementing AI in medical practice” because the predicted improvements in efficiency and accuracy would be nullified by the loss of trustworthiness in the process if explainability were to be given up or reduced to transparency (Duran and Jongsma 2021: 331).

We agree with this point of view yet we would like to make a different contribution to this debate. First, we believe that transparency consists of different “parts” or elements and that a specific part of transparency is fundamental to explainability^8^. More precisely, we argue that *semantic transparency* may address a significant aspect of the problem of opacity. An absence of opacity not only presupposes transparency at the level of *how* symbols and data are *handled* by the AI device, but it necessitates that *exactly what* those symbols and data *represent* be clear and transparent. Therefore, by semantic transparency we refer to the clear and unambiguous usage of terms handled by the CDSS. This forms a crucial element of semantic transparency, the absence of which may serve to undermine any subsequent efforts to provide other forms of transparency, and undermines accuracy, which we argue can provide justification for responsible use of CDSS.

As we explain further in this Section, if the *terminology* used to classify the information that trained an algorithm is unclear, conflated, or insufficiently precise, it will be impossible to obtain transparency at any later stage. Given the foundational nature of the classification of training data, semantic opacity arising from imprecise or conflated terminology at this stage would be extremely difficult, if not impossible, to untangle at a later stage for the purposes of transparency. Therefore, transparency is necessary at this semantic level. In terms of clinical implications, a failure to incorporate semantic transparency can affect both: 1) the ability to understand how an output should be translated into action (i.e., what clinical intervention is advisable); and 2) the degree of accuracy (within a generally accurate range) that can be achieved with the output (i.e., how narrow the range of reliable accuracy is). In this way, semantic transparency is an essential element of reliability needed to support the responsible use of CDSS, for otherwise the actual inner workings of the recommendation system will remain largely unknown to the physician regardless of subsequent reductions in opacity. Therefore, we argue that semantic transparency should be a non-negotiable requirement for transparency in the context of using AI for CDSS, because a lack of this foundational transparency could ultimately undermine the principal value of using an AI device at all.

Unfortunately, the importance of semantic transparency with regard to terminology of both input and output parameters and concepts, is often neglected. If the same input or output symbol within the algorithm can represent different items, or different interpretations of an item, it becomes unclear what exactly is being handled by the algorithm, and different users (who explain the working of the decision to themselves) may have different interpretations of what has been done and what the result is. As philosopher of science Wolfgang Pietsch rightly notes, one of the essential conditions for achieving successful prediction based on data is that “the vocabulary is well chosen, meaning that the parameters are stable causal categories” (Pietsch 2015: 910). Transparency at the semantic level means that the definitions and their operationalization in the algorithm should be transparent (i.e., clear and unequivocal at the semantic level). Pietsch’s requirement of “stable causal categories” refers to the fact that the definition of these parameters should be stable over time, and thus fixed and unchanging, so that any deviations from this requirement over time can be detected.

However, lack of basic semantic transparency is a widespread problem in decision support systems used in clinical medicine. We can illustrate this with an example from the field of nephrology. Acute Kidney Injury (AKI) is a clinical concept indicating that the kidneys are damaged and will rapidly decline in function. Depending on the definition used, this decline can range from rather benign to a complete loss of function, resulting in the accumulation of water and toxins, potentially leading to the death of the patient. Kidney function can to some extent be replaced by extracorporeal renal replacement therapy (RRT). While RRT can be lifesaving, it is invasive and can have life-threatening side-effects such as bleeding, severe electrolyte disorders or low blood pressure. To date, there is no curative treatment for AKI, so there is a lot of focus on algorithm-based automated prediction and early detection in order to avoid progression to AKI.

The correct evaluation and implementation of such algorithms, however, is hampered by an absence of semantic transparency in the use of *many different definitions* of AKI. A review of algorithm-based prediction models for AKI by Van Acker et al. (2021) found that 44 different definitions were used for AKI. Most of these prediction models claim to predict AKI as defined by the widely accepted Kidney Disease Improving Global Outcomes (KDIGO) initiative (Fliser et al., 2012). However, in reality they use *different interpretations* of this definition, which may even substantially differ from the original KDIGO definition. For example, most interpretations neglect the criterion of urinary output, although this is the most powerful prognosticator in the KDIGO definition (Van Acker et al., 2021). As a consequence, the end user cannot truly know exactly *what* is understood by the algorithm-predicted condition labelled as AKI, and how that label should be coupled to possible interventions. *Transparency of prediction algorithms* for AKI requires that we can know *precisely* which definition of AKI was used, and, as a result, understand the implications for intervention. Transparency on the precise definition of AKI used in the algorithm requires in-depth detail not only regarding the definition itself, but also on the exact *operationalization* of that definition into computer language. Indeed, even when the KDIGO definition is correctly used, differences in operationalization might result in differences in the incidence and prognostic value of the label AKI. For example, “patient weight” could be the real, measured weight of the patient, an estimated weight, or an ideal weight for a person of that age and gender and “during 12 h” can be interpreted as “in every hour for 12 consecutive hours” or “over a 12-h period <6 ml/kg.” All these differences in operationalization have a substantial impact on the meaning of the label “AKI” that is provided as an interpretation of the data by the algorithm.

Studies on *interventions* for AKI yield different and contradictory results. This problematic finding is most likely related to the fact that, as mentioned above, different and frequently unspecified definitions are used for AKI, such that, in reality, *different* conditions are being investigated in those intervention studies. Similar instances of conflation, imprecision, and opacity can be found in other fields of medicine, as well (see e.g., Steyaert et al., 2019).

## 3 The importance of explainability in medicine: Accuracy of the recommendation and scientific explanation

It is necessary to engage with various *normative* issues in order to address the following important questions with regard to the specific context of clinical medicine: When and why does explainability matter in medicine? What kind of explainability is necessary in order to reach a responsible use of AI?

Some commentators insist that an elaboration of the *need* for explanation is necessary, and that *the reasons identified for demanding explainability determine what is required to achieve it and what is meant by the term* (Markus et al., 2021: 4). According to Markus et al. (2021), “Given that clinical practice presents a range of circumstances that have different needs regarding explainability, different properties of explainability can be traded off depending on the reason explainability is needed” (Markus et al., 2021: 7). They distinguish three reasons why explainability can be useful (Markus et al., 2021: 4):

(1) “to assist in verifying (or improving) other model desiderata” (e.g., fairness, legality, and ethicality);
(2) to manage social interaction (“to create a shared meaning of the decision-making process” and to justify decisions towards colleagues and patients); and
(3) to discover new insights to guide future research.

They argue that clustering explainable AI (XAI) systems on the basis of need guides determinations about explainability given that need informs “the relative importance of the properties of explainability and thus influences the design choice of explainable AI systems” (Markus et al., 2021: 4).

Adadi and Berrada (2018) identify four motivations for explainability:

(1) to justify decisions;
(2) to enable user control;
(3) to improve models; and
(4) to gain new insights.

It is noteworthy that the lists of motivations for explainability offered by Markus and others and by Adadi and Berrada (2018) both include *the need to garner “new insights.”* As we explain below, this need may be a compelling motivation for explainability in CDSS.

**FIGURE 1:**
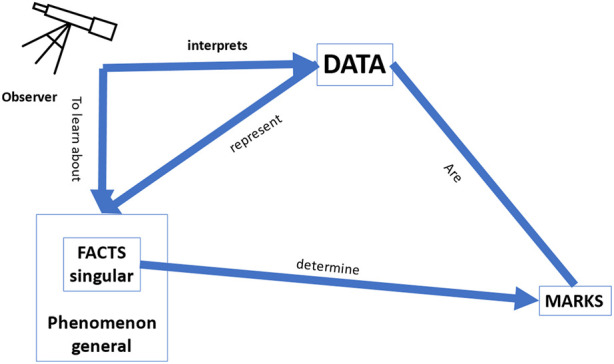
Pietsch’s representation of data (adapted from Pietsch (2021): 11).

In Pietsch’s (visual) representation of the notion of “data” in his book *Big Data* (Pietsch 2021: 11) (Figure 1), he conveys the epistemological importance of data and summarizes the most important (epistemological) aspects of “data” as follows:

“Data are marks on a physical medium … that are meaningfully (i.e., causally or definitionally) related with certain singular facts belonging to a phenomenon of interest. If the data are correctly interpreted in terms of the relationship that they have with those facts, then the data constitute evidence for those facts and thus the phenomenon of interest” (Pietsch 2021: 12).

**FIGURE 2:**
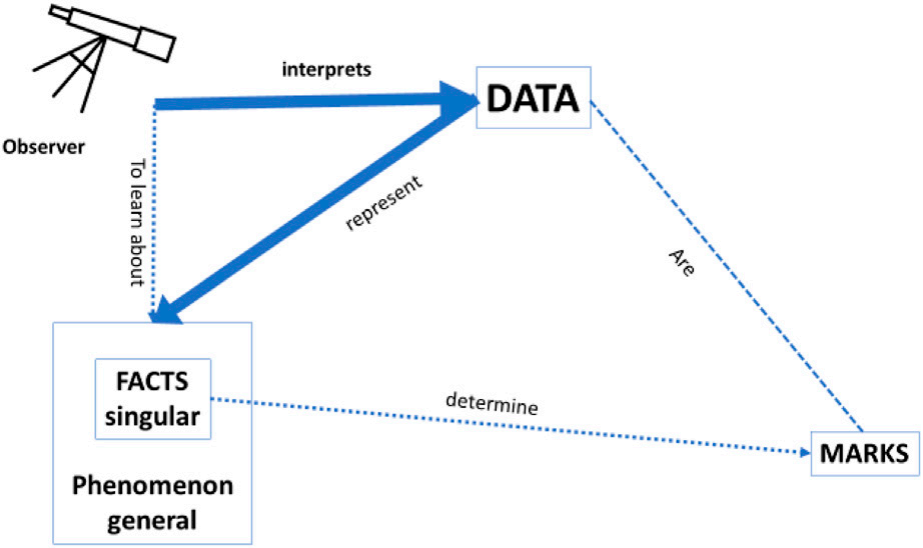
Accuracy: how well the data interpretation (i.e., classification/prediction) by the observer represents the facts/phenomenon. In this setting, it is irrelevant whether the data are causally related to the facts/phenomenon and there is no need for a predefined model.

Bearing in mind this general framework of any data that may provide knowledge about the world, we would like to focus on the specific context of *clinical medicine* where data may provide knowledge about health and disease. Imagine the situation of a clinician in a busy Intensive Care Unit, where an (AI or other) observer gives the clinician her interpretation of the available data. The clinician most likely will only be interested in the *accuracy* of how well this interpretation represents the facts and the phenomenon, and that *will suffice as an “explanation”* to justify the acceptance of an advice (see Figure 2 below). As such, *the accuracy of the recommendation provides an explanation in line with one of the needs* identified by Adadi and Berrada (2018), as mentioned earlier in this Section, viz. the need of “justifying decisions”: the recommendation of the AI-based CDSS is justifiable because it is deemed to be sufficiently accurate. We should point out that even if the framework in which these recommendations was based would turn out to be “wrong” or misguided, the physician would still be justified in using the CDSS if it would be more accurate than any other tool available to them. It is also important to observe that, as can be seen in the figure, there is no need for a causal relation between the data and the recommendation, as long as the accuracy of the CDSS is better than that of any other tool.

Furthermore, the same would be the case for *a patient who was being informed by her physician/nurse about possible interventions*: the patient would like to understand *how* this physician or nurse has linked her data to other data (in other words, how her data were classified) in order to draw the proposed conclusions. For example, to link the data, the physician might have relied on information from an RCT showing that patients with the same condition have the highest probability of having outcome X if they do Y rather than Z. This justification can thus also assist shared decision making, and so corresponds with other reasons why explainability can be needed, e.g., “to manage social interaction and to create a shared meaning of the decision-making process,” “to justify decisions towards colleagues and patients,” and “to enable user control” [cf. the lists above of Markus et al. and of Adadi and Berrada (2018)].

Accordingly, we would argue that in clinical decision support systems, knowledge on the *accuracy of the recommendation is an essential part of explainability*, especially for the clinical practitioner and the patient confronted with an acute task of decision making. By accuracy of the recommendation we mean that in daily clinical practice, it is sufficient most of the time to have *an explanation that provides enough justification to (not) do something*. However, *accuracy by itself is insufficient to satisfy all the needs-based criteria*. It provides a justification for using the CDSS in the whole group of patients, but cannot identify possible fallacies of the CDSS in relation to an individual patient, as it cannot provide (causal) insights in the process. This may have serious adverse consequences in individual (exceptional) cases, or in conditions in which the model becomes unstable.

To avoid such adverse consequences, explanation should be about *why* a physician or a CDSS *classified* a patient as belonging to a particular group*.* Indeed, as noted by Duran, accuracy of prediction or classification does not explain the true relations between the data and the outcome. Duran (2021: 3) argues, convincingly in our view, that explanations must be distinguished from “other epistemic functions, such as predictions, classifications, and descriptions” and that “much of what today is taken to be XAI are, in fact, classifications and predictions” whereas “scientific explanations provide a particular type of valuable information, one that grows our understanding of *why* a given output is the case, rather than organizing our knowledge and possibly forecasting new cases.”

Importantly, in some cases, causal models regarding underlying mechanisms might lead the physician to make wrong clinical decisions. For example, dopamine induces diuresis and, on the basis of this physiological property, it used to be administered to patients to prevent AKI; however, we now know from RCTs that the use of dopamine is associated with higher mortality and more AKI, so this practice has been abandoned. While the explanation based on physiology would lead a physician to use dopamine, the “explanation” provided by RCT data would discourage the physician from doing so. This stresses, once again, the importance of taking into account the *aim* of the explanation when defining what can be seen as explanation.

Nevertheless, incorrect predictions are more likely to be avoided (and accuracy is thus more likely to be improved) if one can rely on a *model of the world* rather than on mere associations between input and output. Errors can result, for example, from so-called *tank problems*, where the algorithm bases its recommendation on data that do not have any relevant relation to the facts they represent, but only an—often obscure—association with those facts. (Zech et al., 2018).

**FIGURE 3:**
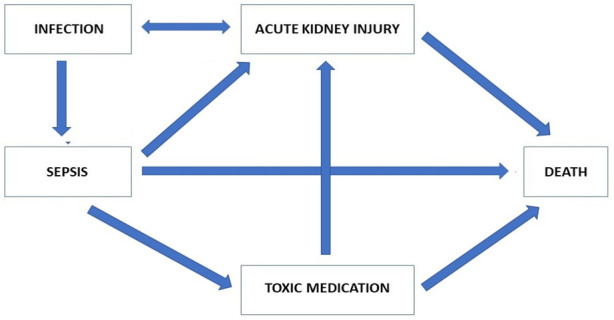
Modelling of the most important factors involved in the relationship between AKI and mortality.

What matters most for the *daily practice* of clinicians is “classification.” Physicians usually work by classifying a patient into a certain group. In fact, clinical guidelines are generally developed to facilitate this kind of patient classification. In this setting, it is not necessary that the data are causally linked to the outcome, as long as the final classification is accurate. However, a classification is not the same as an explanation in the sense of understanding *why* certain things happen the way they do. It is learning about the world by association, not by making a model. Nonetheless, in order to advance medicine and reduce future errors, the effort to continue seeking to understand the *why*, i.e., the causal mechanisms, is essential (see Figure 3 below). Understanding causal relations in the data might improve accuracy, as this would avoid recommendations based on non-causal correlations, a weakness that is lurking in many deep learning systems. As the medical community has a duty to provide the best care possible, it is justified to use a CDSS with an accuracy higher than that of physicians. The medical community also has a duty, moreover, to improve accuracy by trying to better understand causal relations in the data and thus improving the model, and thereby improving the accuracy of the CDSS in the future.

*Scientific explanation* (see Duran and Jongsma, 2021) of a prediction model for AKI would require a clarification of *whether* and *why* a given factor has a causal contribution to the development of AKI, and how much of the emergence of AKI *and* the associated mortality is *attributable* to that factor. Such an explanation is even more important since the fact that AKI is *associated* with mortality does not necessarily imply that avoiding AKI would decrease mortality. A scientific explanation would be required for understanding the process as well as for being able to develop strategies to avoid or minimise this factor. Depending on the extent to which the likelihood of AKI and of mortality are attributable to this factor, that clarification might thus also allow for a reduction of the probability of AKI and thus mortality.

From Figure 3, we can see that AKI in patients with sepsis can be linked to mortality; however, it is equally apparent that AKI is associated with many other factors, which in themselves are associated with mortality. *Explaining* the causal pathway is an essential step to improving the outcome for these patients. Indeed, even if a golden bullet were invented that would totally prevent patients with sepsis from developing AKI, many other pathways leading to death could still exist. As long as the relative impact of the direct association between sepsis and death and the effect of AKI as a consequence of sepsis is not *explained*, it would not be possible to predict the change in mortality of sepsis with AKI by treatment with the golden bullet.

**FIGURE 4:**
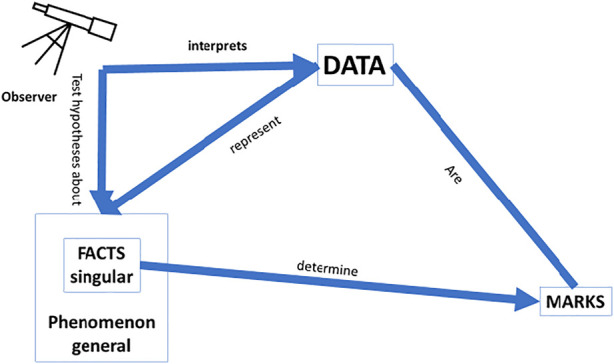
Scientific explanation: This form of explanation leads to a better understanding of the world; operationalisation of the data needs to be in line with a causal relation between the facts and the marks; a model that can be tested as a hypothesis is needed to ascertain whether or not the data fit the model; and the facts need to be causally related to the phenomenon they instantiate.

For a scientifically explainable AI (sXAI) for CDSS, one would need an interpretable predictor that helps one *understand* how the phenomenon that is determined by the facts, which are themselves potentially described by the data, causally relates to the *phenomena* (Figure 4). Such understanding could rely on knowledge regarding physiology, counterfactual experiments, etc. Such understanding could enhance and improve the classifications made by physicians in future cases, for it would also allow for generalization of the current data and situations to cases outside the current dataset, precisely because a correct understanding of the *why* would then be available.

In daily life a sufficient explanation to a physician is *an explanation that gives her enough justification to do or not do something*. One simply could not work as a physician if one sought to understand the underlying pathophysiological mechanisms all the time. A physician wants to classify a patient (intervention X will work because the patient belongs to class Y), to achieve a justifiable balance between accuracy and having time to treat all the patients who need her help. However, *in order to improve the accuracy of medical decisions in the longer term*, we need a better understanding of the phenomenon based on causal models. We will now proceed to take a closer look at this.

## 4 The importance of explainability in medicine: Big data and scientific explanation

Our argumentation so far highlights accuracy as an essential part of explainability of the use of CDSS, but at the same time supports a demand for greater understanding of causality as essential to the advancement of medicine, not least because increased understanding of causal factors is expected to result in increased accuracy. Therefore, we argue that prioritizing accuracy implies that one has to pay attention to causal mechanisms to ensure accuracy in the long term.”

Accordingly, if, in order to ensure accuracy in the long-run, we need to be able to make models of causal mechanisms*, a central question remains of whether deep learning and Big Data approaches are helpful at all to answer “why” questions*. If the answer to this question is “no” it seems that it would be impossible for CDSS based on big data or deep learning ever to become completely 100% accurate. In what follows we use the work of Wolfgang Pietsch to try to answer this question.

As shown in Figure 2, in a deep learning approach without a predefined theoretical model, the interpretation of the data by the observer is only assessed by the accuracy of how well the data predict the facts and the phenomenon, but there is no guarantee of a *causal* relation between the data and the facts, and between the facts and the phenomenon. In the approach of Pietsch, a causal relation would be could be uncovered if all relevant factors are included in the dataset, the background conditions are stable, and all potentially relevant combinations of factors are present. (Pietsch, 2015) In such a setting, a causal relation can be derived between the data and the phenomena by so-called *variational induction*. Pietsch stresses that the variety of evidence is crucial for variational inductivism: “confirmation … increases … with observing as many different situations in terms of changing circumstances as possible” (Pietsch 2021: 30). However, in clinical practice, one can never be certain that all relevant factors are represented in the dataset in all potentially possible combinations. Therefore, whereas the claim of causal conclusions based on variational induction is correct in theory, in practice it does not hold. This is an important nuance, all too often neglected in Big Data analysis. It explains why, for example, all the reports in the literature of successful applications of deep learning in the context of medicine—with the term “successful” referring to cases where the classification skills of the algorithm were comparable to those of experienced clinicians—concern cases where the number of data used to train the system was very high, and/or with a strongly restricted focus (e.g., to the question “diabetic retinopathy or not” in patients with diabetes, and not “what is the eye disease in this person” in the general population), precisely to ensure that all potential combinations of relevant factors can be present in the data set.

In other words, data can teach us something about the world *via* the association between the data and the facts, but only in the *specific context* of where, how and when the data were generated, and not about what would happen in a *counterfactual* world where some of the parameters are different. The fact that the relations between the data and the facts and the facts and the phenomenon are only associational does not preclude accurate predictions as long as the circumstances in which the predictions are made remain identical. However, as soon as the circumstances change (e.g., if the algorithm is used in a different hospital), the algorithm might become biased as the relation between the facts and the data in the original algorithm was not causal. If, for example, one of the data points that determined the classification by an algorithm was the type of X-ray machine used, this is of course not causally related to the type of lung disease that needs to be diagnosed. The only way to get out of this conundrum is to have a theoretical framework of the world, as this would identify which (combination) of data elements are necessary to accept that “all potentially relevant factors are included in the dataset, the background conditions are stable, and all potentially relevant combinations of factors are present,” as requested by Pietsch (2015). Indeed, as noted earlier, in a clinical setting, the theoretically correct concept of variational induction allowing causal conclusion, only holds when a pre-specified model of the world is used to guarantee that all potentially relevant factors are present in the dataset.

In clinical settings big data sets never contain all the relevant data, and, given that inclusion of irrelevant data can lead to “tank problems,” it is essential to *build a model* of the condition to allow for generalisation. The only way to achieve such a model is by *exploring causal relations* between the data and the observed phenomena, i.e., by scientific explanation. Therefore, to ensure the accuracy of our interventions in the long term, we have to continuously improve our theoretical models by studying causal mechanisms.

However, in the daily life of physicians, understanding such causal relations is not *per se* sufficient to select a certain intervention. There will always be a need to validate whether in reality the assumed causal relations will lead to improved outcomes, as is exemplified by the dopamine case. Scientific explanation *alone* cannot replace accuracy as a justification for using a certain intervention if the intervention is not tested in clinical trials. Therefore, the view of Pietsch needs further nuance: explanation understood as clarifying causal mechanisms and/or development of a model is necessary to improve accuracy of an existing CDSS, but is on itself insufficient to justify the use of the new CDSS. In order to justify this use, the accuracy of the improved CDSS should be better than that of human physicians, the previous version of the CDSS or other tools for decision making in the context at hand, making accuracy essential part of the explanation of why it is justified to use the CDSS.

## 5 Concluding remarks

The potential of AI to serve as a valuable aid in medical decision-making is significant but is still some distance away on the horizon. The acceptance and integration of AI-driven systems in everyday clinical practice depends on multiple factors. In this paper, we have focused on what kind of explainability is necessary to use CDSS responsibly in a clinical context. We identified three factors that are crucial to explainability in the context of responsible use of CDSS.

First, we identified semantic transparency, a specific type of transparency, as a critical component of transparency’s contribution to explainability, and an essential element of what is required for responsible use of AI systems in the context of CDSS. Second, as some scholars have noted, the importance of explainability varies according to need. We have found that, in daily clinical practice, most of the time, accuracy should and does serve as a necessary and sufficient basis for responsible use of AI in CDSS by physicians. Third, building on Duran’s (2021) case for the need for scientific explanation, we have argued that in order to improve accuracy in the longer term, and thus to reduce the incidence of interventions that negatively affect the survival and health of future patients, understanding underlying causal mechanisms is necessary. When we understand the underlying mechanisms, we can understand why some patients respond to particular treatments and others do not. Scientific explanation is thus necessary to enhance accuracy. However, understanding a causal mechanism of a disease, a diagnostic test, or an intervention does not necessarily lead to improved outcomes when acted upon in the clinical reality. This can only be achieved with clinical trials. Therefore, scientific explanation is *in itself* insufficient to justify clinical actions.

We support the view that transparency is of limited value as a surrogate for explainability (Markus et al., 2021: 3). Nevertheless, we have identified semantic transparency as fundamental to explainability, in that *semantic transparency* may address a significant aspect of the problem of opacity. That is, given the foundational nature of the classification of training data, semantic opacity arising from imprecise or conflated terminology at this stage would be extremely difficult, if not impossible, to untangle at a later stage for the purposes of transparency. However, lack of basic semantic transparency is a widespread problem in decision support systems used in clinical medicine. For this reason, we stress this type of transparency as essential to explainability for two reasons: 1) it provides a specific type of accuracy that is necessary for the responsible use of CDSS; and 2) given that semantic transparency yields precision, it furthers the ability to derive causal explanations, which in turn, leads to increased accuracy. Understanding causal relations in the data can improve accuracy, as this would avoid recommendations based on non-causal correlations, a weakness that is lurking in many deep learning systems. However, in the daily life of physicians, understanding such causal relations is not *per se* sufficient to select a certain intervention. There will always be a need to validate (by means of Randomized Controlled Trials) whether in reality the assumed causal relations will lead to improved outcomes.

Our goal should be to create support systems for clinical decision-making that give the best possible outcome as much of the time as possible; that are as good as they can be until the *why* is understood; that actively “seek” causality; that are compatible with subsequent value-based choices; and that are open to improvement^9^. We fully concur with London’s (London 2019: 20) recommendation that “regulatory practices should establish procedures that limit the use of machine learning systems to specific tasks for which their accuracy and reliability have been empirically validated.”

London also rightly observes that the pathophysiology of disease is often uncertain and the mechanisms through which interventions work is frequently not known or, if known, not well understood (London 2019: 17). However, we would submit that this is a reason to strive more, rather than less, for understanding and explanation. As Aristotle observed and London has carried forward in his work, medicine is both a science and an art. We take the view that it is indeed both, but that although accuracy may be prioritized with regard to the patient in the clinic today, there are practical and pressing reasons to attend to causal knowledge in order to best serve tomorrow’s patient.

## Data Availability

The original contributions presented in the study are included in the article/supplementary material, further inquiries can be directed to the corresponding author.

## Footnotes

1 There are also other reasons why some have argued we need explainable AI. It is argued, for instance, that explainable AI to avoid widespread discrimination by AI (Gerke, 2021). Although we are aware of these arguments, in this paper we will focus on the need arising from the black box character of CDSS. Nevertheless, we believe the criticism we offer Section 2 of our paper is also relevant to these arguments.

2 See https://www.bbvaopenmind.com/en/science/leading-figures/james-lind-and-scurvy-the-first-clinical-trial-in-history/ [last accessed 8 August 2022].

3 We will not be looking at explainability concerning AI in a non-medical context. We hypothesize, however, that several differences exist between explainability of AI in the clinical-medical setting as opposed to other settings. First, the decision of an AI in a medical context cannot be evaluated by merely creating *transparency* regarding the factors that drove the decision, because the relationship between input and output is less clear in a medical context. In other domains there is either a clear and established relation between input and output, or, when such a relation is unknown, it can easily be tested whether or not the use of the algorithm would consistently lead to the desired result even if all other factors were to be varied. Second, medical decisions inevitably reflect values. Even seemingly objective decisions, for example, the thresholds selected to steer medical decisions, in reality reflect one or several values. In this paper, however, we will not focus on these differences.

4 See also Berner and La Lande (2016).

5 In recent years there has been an interesting debate concerning the status and nature of causality within the field of medicine (Kincaid, 2008; Thompson, 2011; Illari & Russo, 2014). In this paper we will focus on the question whether CDSS is able to meet the demands of clinical practice, assuming that the modeling of causal relations plays at least *some* role in clinical practice.

6 Berkeley sociologist and computer scientist Jenna Burrell makes an important distinction between three forms of opacity: opacity as intentional corporate or state secrecy (in order to maintain a competitive advantage); opacity as technical illiteracy (because code writing and reading are specialist skills); and opacity that arises from the characteristics of machine learning algorithms, more specifically from “the mismatch between mathematical procedures of machine learning algorithms and human styles of semantic interpretation” (Burrell 2016: 3). Even though all three forms can be relevant for the context of clinical medicine, we will only be concerned with the third form.

7 According to Duran and Jongsma, this need not worry us too much because the outcomes of medical AI *can* be trustworthy and black box algorithms *can* be reliable, provided that certain epistemic conditions are met, viz. the conditions entailed by the framework of “computational reliabilism” that they propose (Duran and Jongsma 2021: 332; Duran and Formanek 2018). Although they argue that this framework, which does not require transparency, provides “reasonable levels of confidence about the results of opaque algorithms,” this claim does not imply that opaque algorithms should be used without any restrictions, as the appropriateness of their use in the context of medicine depends on many factors that are related to ethics rather than epistemology (Duran and Jongsma 2021: 330).

8 By “fundamental” we mean that although semantic transparency is not a sufficient condition of explainability, it is a necessary one.

9 The latter being something which may be hindered or prevented by proprietary regimes such as patents and trademarks. This is a highly important topic, but we cannot elaborate on it here.
